# Psychoactive substances: novel molecular insights and therapeutic potential for Alzheimer's disease

**DOI:** 10.1186/s40035-025-00498-1

**Published:** 2025-07-25

**Authors:** Chonglei Fu, Xuehui Li, Xiaoxing Liu, Yongbo Zheng, Xiangxian Ma, Bo Zhang, Si Chen, Lingming Hu, Sizhen Su, Xiaokun Wang, Jie Sun, Qingqing Yin, Yumei Wang, Xiaolong Fu, Yanping Bao, Yanxue Xue, Tao Xin, Lin Lu, Guichang Zou

**Affiliations:** 1 Department of Clinical Psychology, Shandong Provincial Hospital Affiliated to Shandong First Medical University, Jinan, 250021 China; 2 Institute of Brain Science and Brain-Inspired Research, Shandong First Medical University and Shandong Academy of Medical Sciences, Jinan, 250117 China; 3 Shandong Institute of Brain Science and Brain-Inspired Research, Jinan, 250117 China; 4 Hospital for Skin Diseases, Shandong First Medical University, Jinan, 250021 China; 5 Shandong Provincial Institute of Dermatology and Venereology, Shandong Academy of Medical Sciences, Jinan, 250021 China; 6 National Clinical Research Center for Mental Disorders (Peking University Sixth Hospital), Peking University Sixth Hospital, Peking University Institute of Mental Health, Beijing, 100191 China; 7 Peking-Tsinghua Centre for Life Sciences and PKU-IDG/McGovern Institute for Brain Research, Peking University, Beijing, 100871 China; 8 National Institute on Drug Dependence, Beijing Key Laboratory of Drug Dependence, Peking University, Beijing, 100191 China; 9 Pain Medicine Center, Peking University Third Hospital, Beijing, 100191 China; 10 Department of Geriatric Neurology, Shandong Provincial Hospital Affiliated to Shandong First Medical University, Jinan, 250117 China; 11 Medical Science and Technology Innovation Center, Shandong Provincial Hospital, Shandong First Medical University and Shandong Academy of Medical Sciences, Jinan, 250117 China; 12 Department of Neurosurgery, The First Affiliated Hospital of Shandong First Medical University and Shandong Provincial Qianfoshan Hospital, Jinan, 250013 China

**Keywords:** Alzheimer's disease, Psychoactive substances, Molecular insights, Therapeutic potential, Challenges

## Abstract

Alzheimer's disease (AD) is a major neurodegenerative disorder that causes severe cognitive decline and poses a significant burden on global health systems. Despite extensive research, effective treatments to stop neurodegeneration or promote neuronal regeneration in AD remain elusive. Psychoactive substances as modulators of neurophysiological functions, have received increased attention in research. The main psychoactive agents, such as central nervous system depressants and stimulants, cannabinoids, psychedelics, opioids and ketamine, are being explored for their abilities to enhance learning and cognitive performance and potential neurorestorative functions. In this paper, we review the molecular mechanisms and therapeutic potential of psychoactive substances in AD, in the aim to guide future research directions.

## Background

AD is a neurodegenerative disorder characterized by dementia [1, 2]. According to the World Health Organization (WHO), over 50 million people worldwide suffer from AD and the number is expected to double by 2050 due to the global aging trend [3, 4]. The economic costs of AD are substantial, with annual global costs exceeding those of heart diseases, cancer and stroke, highlighting the urgent need for effective treatment strategies [5]. Despite advances in the understanding of complex AD pathogenesis such as amyloid-beta (Aβ) aggregation and tau protein pathology, the high rate of failure in clinical trials has hindered the development of disease-modifying therapies [6]. Current FDA-approved treatments only provide symptom relief. Although monoclonal antibodies targeting Aβ have shown promise, their efficacy has been controversial and less than satisfactory [7–10]. This highlights the need for novel therapeutic strategies targeting the molecular basis of AD.

Psychoactive substances are emerging as potential therapeutic agents for AD due to their different pharmacological characteristics and ability to modulate neural networks [11–13]. Their potential to regulate brain functions and improve cognition offers new perspectives on addressing the multifaceted nature of AD. We searched PubMed, Science Direct and Web of Science databases for articles exploring the relationship between psychoactive substances and AD, published between 2000 and 2025. The keywords include Alzheimer's disease, psychoactive substances, benzodiazepines, therapeutic potential as well as other relevant terms. The inclusion criteria are original studies and review articles related to both AD and psychoactive substances. Literatures published in non-English languages were excluded. This review focuses on the molecular targets and mechanisms of action of psychoactive substances in AD and discusses their therapeutic potential and challenges in development and application.

## Molecular insights into AD

**Fig. 1:**
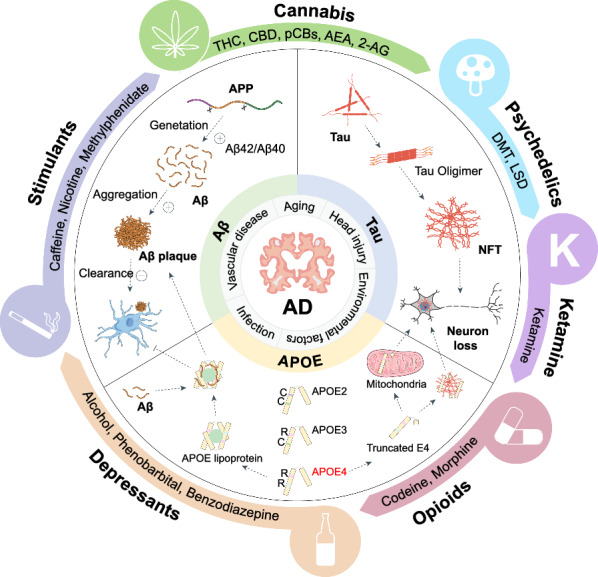
Schematic of molecular insights into AD etiology and psychoactive substances. Excessive Aβ aggregation, tau protein phosphorylation and APOE are the main characteristics of AD pathogenesis. Other risk factors include aging, head injury, vascular disease, infection and environmental factors. Briefly, altered APP processing leads to overproduction of Aβ, particularly the more aggregable isoform Aβ42, resulting in an increased Aβ42/Aβ40 ratio. Tau protein hyperphosphorylation impairs its microtubule-stabilizing function, leading to its aggregation into nerve fiber knots and finally causing neuronal death and damaging neural networks. APOE4 also significantly increases the risk of AD. Structurally intact APOE4 prevents Aβ clearance and promotes Aβ deposition, while structurally incomplete APOE4 always acts as a neurotoxic factor and causes neuronal death via promoting tau deposition and mitochondrial dysfunction. The main psychoactive substances showing potential therapeutic efficacy in AD include CNS depressants and stimulants, cannabis, psychedelics and opioids

AD is a progressive and persistent neurodegenerative disease characterized by cognitive decline, the most prominent manifestation of which is progressive memory loss [14, 15]. Pathologically, AD is characterized by Aβ plaques, neurofibrillary tangles (NFTs), neuronal death and gliosis, accompanied by various neuroinflammation and synaptic function alterations [16, 17]. Indeed, there is considerable evidence suggesting that AD is a multifactorial disease, with the top three factors being Aβ aggregation, tau protein phosphorylation and apolipoprotein E (APOE) [18–24]. Additionally, other risk factors such as aging, head injury, vascular disease, infection and environmental factors also play a critical role in the onset of AD [25] **(**Fig. 1)**.**

## Aβ, tau hypothesis and APOE

The Aβ hypothesis posits that excessive Aβ, particularly the aggregation-prone Aβ42, is a key driver of AD. Supporting this hypothesis, genetic mutations in amyloid precursor protein (*APP*) and presenilin (*PSEN1/2*) genes lead to increased Aβ production and early-onset hereditary AD forms [26–28]. Aβ oligomers, which interact with neuronal receptors and disrupt calcium homeostasis, are implicated in synaptic dysfunction and neuronal toxicity [29]. Despite the amyloid cascade hypothesis, therapeutic strategies targeting Aβ alone have shown limited efficacy in clinical settings, suggesting the complexity of AD pathogenesis extends beyond Aβ [29–31].

The tau hypothesis focuses on the role of hyperphosphorylated tau protein in NFT formation, a hallmark of AD. Tau stabilizes microtubules and its dysfunction and subsequent aggregation is linked to synaptic and neuronal degeneration [32, 33]. The phosphorylation of tau protein at sites such as S396 and T231 has been demonstrated to promote tau aggregation and serves as potential biomarkers for AD [34]. Regulation of tau phosphorylation by kinases such as glycogen synthase kinase 3 beta and cyclin-dependent kinase 5 is a critical therapeutic target [35].

*APOE* is the most common genetic risk factor for AD and has become an important therapeutic target. APOE is a protein containing 299 amino acids and is mainly produced by astrocytes and microglia [36]. APOE is crucial for the catabolism of triglyceride-rich lipoproteins and interacts with various cell surface receptors to regulate the transport of lipids (such as cholesterol) into neurons. To date, three subtypes of APOE have been identified: APOE2 (Cys112 and Cys158), APOE3 (Cys112 and Arg158) and APOE4 (Arg112 and Arg158) [37]. The amino acid differences among subtypes are small, but such differences can affect the total charge and structure of APOE, thereby influencing its binding to cell receptors and lipoprotein particles and may affect its stability and production/clearance rate [36]. Increasing evidence indicates that APOE4 increases the risk of AD and approximately 50% of sporadic AD cases can be attributed to this allele. Specifically, individuals carrying one copy of the *APOE4* allele have a higher risk of developing AD compared to non-carriers, and those with two copies exhibit an even greater risk, highlighting the dose-dependent contribution of *APOE4* to AD susceptibility. On the contrary, *APOE2* reduces the risk of AD [38, 39]. APOE4 has no effect on the synthesis of Aβ, but affects the clearance and deposition of Aβ plaques. Therefore, the subtypes and quantities of APOE in the CNS are crucial factors for the pathogenesis of AD.

## Other risk factors

Beyond Aβ, tau and *APOE*, other factors such as aging, genetic mutations and environmental influences can contribute to AD etiology. Early-onset AD is often familial and linked to dominant genes like *APP* and *PSEN1/2*, while late-onset AD is more common and associated with aging-related factors [40].

The interplay between genetic predisposition and environmental factors, as well as their impact on oxidative stress and inflammation, underscores the need for targeted and individualized therapeutic approaches. Head injuries, vascular disease, infection and environmental factors may induce oxidative stress and inflammation and increase the AD risk. Chronic CNS infections such as infections by herpes simplex virus, spirochaetal bacteria and *Chlamydophila pneumonia*, promote inflammatory responses and Aβ deposition, which then lead to neuronal damage and calcium imbalance, ultimately causing cognitive deterioration [41–43]. In addition, long-term exposure to air pollutants induces oxidative stress, neuroinflammation and aberrant accumulation of phosphorylated tau and Aβ protein, which also contribute to cognitive function impairment [44, 45]. Considering the multifactorial pathogenesis of AD, intervention and treatment strategies for AD should be individually designed to address the diverse pathological processes.

## New approaches and targets

In recent years, Cryo-EM-based studies have successfully resolved the structure of human brain-derived Aβ42 filaments, identifying two S-shaped protofilament folds that give rise to two types of filaments. Type I filaments are predominantly found in the brains of sporadic AD patients, whereas type II filaments are found in the brains of patients with familial AD [46]. This provides a theoretical foundation for the development of more precise Aβ-targeting drugs.

In AD, a variety of mechanisms of cell death has been observed in AD, including apoptosis, necrotic apoptosis, inflammatory cell death and ferroptosis, as well as autophagy- and lysosome-related processes, suggesting cell death regulation as a novel treatment strategy for AD [47].

Microglia-related pathogenesis of AD is also a cutting-edge research area. Mononuclear RNA sequencing reports that the ACSL1-positive microglia are most abundant in AD patients with the *APOE4/4* genotype. The conditioned medium of microglial cells, which contains lipid droplets, causes tau phosphorylation and neurotoxicity in an *APOE*-dependent manner [48]. Terminally inflammatory microglia, which represent an exhausted state of inflammatory microglia, are associated with AD risk and pathology in *APOE4* carriers and the elderly [49]. This indicates a potential association between genetic risk factors for AD and microglial lipid droplet accumulation and neurotoxicity, which may provide novel therapeutic strategies for AD.

Additionally, the gut-brain axis is also associated with AD and there is evidence that microbial metabolites can influence neuroinflammation and Aβ clearance. Regulating the gut microbiota to promote the production of beneficial metabolites is a promising direction of AD treatment [50–52]. The potential of epigenetic modifications such as DNA methylation and histone acetylation to influence disease progression and response to therapy is another emerging field in AD [53]. Understanding the epigenome in AD may pave the way for personalized treatment that considers an individual's genetic and environmental interactions.

Recent advances in drug development strategies include the use of PROTACs (proteolysis-targeting chimeras), and PPI (protein–protein interaction) modulators [52]. T209S mutation in the *ZDHHC21* gene can enhance the palmitoylation of Src family tyrosine kinases and APP, leading to synaptic dysfunction and cognitive impairment [54]. This finding suggests that *ZDHHC21* and associated protein palmitoylation may be a new target for AD treatment. In addition, another study shows that macrophage stimulating 1 (MST1) activation is positively correlated with Aβ accumulation and promotes neuronal apoptosis through the p53 signaling pathway, ultimately triggering cognitive decline and neurodegenerative changes [55]. This suggests that MST1 and its related signaling pathways may be another potential target for AD treatment. With antioxidant and anti-inflammatory properties, *Mandukparni* (*Centella asiatica Linn* Urban) and Licorice may also reduce oxidative stress and inflammatory response, promote nerve growth and synaptic plasticity in the brains of AD patients, and ultimately improve cognitive function [56, 57]. Both herbs offer natural and multi-targeted prospects for the development of new therapeutic drugs for AD. These advances in cellular and molecular research provide new insights into the pathogenesis of AD and open up possibilities of developing non-traditional therapeutic strategies.

## Current status of treatment

The complex pathogenesis directly results in limited therapeutic agents and a high failure rate of new drug development for AD. During the past decades, most efforts have been focused on exploiting novel strategies against the Aβ protein. However, their efficacy in alleviating symptoms and slowing disease progression is unsatisfactory [33]. Compared with Aβ, tau appears to be a more reliable interventional target due to its close association with cognitive decline in AD. To date, four anti-tau monoclonal antibodies and one anti-tau vaccine have entered phase II clinical trials [58]. Nevertheless, immune therapy still faces numerous challenges, including various adverse effects such as edema and bleeding [59, 60]. Furthermore, blockade by the blood–brain barrier (BBB) necessitates the use of high doses of antibody, which may cause additional unwanted effects [61].

The current main methods for targeting *APOE* to treat AD are as follows. One approach is to develop small molecules or antibodies that can regulate the structure and function of APOE to enhance its neuroprotective effect [62]. Another strategy involves gene therapies to reduce the expression of *APOE4* or promote the expression of neuroprotective *APOE2* [63]. Lipid management is also under exploration because APOE plays a key role in lipid metabolism. By regulating levels of lipids, especially cholesterol, it is possible to improve the function of APOE in lipid transport and Aβ clearance [64]. Preclinical studies have shown that certain compounds can alter the distribution of APOE subtypes, reduce the level of APOE4 and increase the level of APOE2, which may have a positive therapeutic effect on AD [65]. In addition, researchers are developing APOE mimetic peptides to simulate the beneficial effects of APOE on lipid metabolism and synaptic function [66]. However, these therapies are still at early stages and face challenges in clinic, such as in achieving effective brain delivery and regulation of APOE function without adverse reactions.

Some novel therapeutic and pharmacological approaches are currently under development, including cell replacement therapy [67], nano-liposome and exosome delivery systems [68]. In 2024, there are 164 trials and 127 drugs in the AD drug-development pipeline. Of the 164 trials, 34% tested biologics to improve disease, 41% tested small-molecule drugs to improve disease, 10% tested drugs to enhance cognition and 14% tested drugs to treat neuropsychiatric symptoms [69]. It should be noted that despite the ongoing clinical trials, the likelihood of identifying a single cure for AD is low. Upon this, it is of the utmost importance to gain a deeper understanding of the molecular etiology and progression of AD, taking into account both genetic predispositions and environmental factors.

## Therapeutic potential of psychoactive substances

Psychoactive substances are a class of natural or synthesized chemicals that can have significant effects on mental processes such as thinking, emotion and volitional behavior by altering brain function [70]. Based on their properties and neurobiological mechanisms of action, they are typically categorized as CNS depressants and stimulants, cannabis, psychedelics, opioids and ketamine. Active substances have been long used in the treatment of mental disorders and neurological disease, with evidence dating back to the Middle Ages [71, 72]. With the deepening of research into the pathophysiology of neurodegenerative diseases, psychoactive substances have received extensive attention due to their potential neuroprotective effects. In recent years, numerous studies have underscored the role of psychoactive substances in enhancing learning and memory, particularly in the treatment of AD.

### Depressants

CNS depressants are drugs that inhibit the nervous system, including benzodiazepines (BDZs), barbiturates and ethanol [73, 74]. These drugs act on gamma-aminobutyric acid type A receptors (GABA_A_Rs), enhancing the binding of GABA to the receptors and causing sedative, anti-anxiety, hypnosis and anticonvulsant effects [75, 76].

#### BDZs

BDZs are a class of psychoactive drugs known for their rapid depressant effect on the CNS. They are chemically characterized by a benzene ring fused to a diazepine ring. BDZs are commonly synthesized and are not present naturally in plants or animals [77]. They are positive allosteric modulators of GABA_A_Rs, enhancing the inhibitory effect of GABA, reducing neuronal excitability and generating sedative, anti-anxiety, hypnotic, anticonvulsant and muscle-relaxant effects. Diazepam, alprazolam and lorazepam are the most common BDZs and are widely used in various clinical settings to treat anxiety, insomnia, epileptic seizures and alcohol withdrawal [78]. BDZs such as alprazolam, clonazepam, diazepam, estazolam, lorazepam and quazepam, have shown great potential to affect the progression of AD [79, 80]. A recent study indicated that the administration of short-acting BDZs, which typically have a duration of action of several hours, is associated with a reduced Aβ burden in patients diagnosed with AD [81].

Studies in clinical cohorts and rats have suggested that BDZs improve cognitive function in AD by activating inhibitory GABAergic neurons, exerting anti-inflammatory and neuroprotective effects and inhibiting Aβ aggregation and tau protein phosphorylation [82]. Long-term propofol treatment also reduces Aβ production and promotes Aβ clearance by decreasing β-site APP-cleaving enzyme 1 levels and increasing neprilysin levels in the brains of aged mice [83]. The activation of GABA_A_R enhances neurogenesis in mice, while the *APOE4* variant impedes it [84–86]. Notably, although GABA_A_R stimulation is associated with increased neurogenesis, there is still debate on the presence and the degree of adult hippocampal neurogenesis in the human dentate gyrus. Some studies have shown that hippocampal neurogenesis is almost absent in people over the age of seven [87, 88]. Therefore, although neurogenesis promotion has been well established in rodent experiments, it is still unknown whether related drugs can be applied to AD patients. Strong evidence from clinical and basic research suggests that APOE4 promotes the progression of AD by impairing the function of hippocampal GABAergic neurons [84, 85]. Consequently, activation of GABA_A_R by BDZs can effectively alleviate the toxic effects of APOE4 on GABAergic neurons [89]. These findings suggest that BDZs may offer some protective effects against the development and progression of AD.

However, the direct adverse effects of BDZs causing memory impairment and cognitive decline should not be ignored. Preclinical studies in mice have shown that long-term use of BDZs leads to decreased release of neuroprotective brain-derived neurotrophic factor, further accelerates Aβ deposition, reduces synaptic transmission and finally disrupts calcium homeostasis in AD [90]. Additionally, research in rodents has reported that BDZs exacerbate AD progression by increasing tau protein phosphorylation, which consequently causes insulin resistance and compensatory overactivation of GABAergic neurons [91–93]. However, in a population-based longitudinal study involving older adults, no association is found between the highest level of BDZ use and dementia or cognitive decline [94]. A substantial cohort study also shows that there is no association between the use of BDZs and dementia [95]. Although the interlink between BDZs and AD remains controversial, current evidence consistently supports that the GABAergic system is a valuable target for improving cognition and impeding AD development.

#### Ethanol

Ethanol (C_2_H_5_OH), also known as ethyl alcohol or simply alcohol, is a common psychoactive substance that can affect the CNS by producing intoxicating effects at higher doses. It exerts both beneficial and harmful health effects, depending on the amount and the pattern of use [96]. There is a complex relationship between alcohol and dementia. Although epidemiological evidence suggests that moderate levels of ethanol consumption may have a protective effect against Aβ-induced synaptic failure [96], relevant studies are rare and only limited to animals. Notably, the WHO and many other regulatory agencies worldwide announce that no levels of ethanol consumption are safe for human health. Animal studies have shown that binge drinking in adolescents accelerates Aβ production and cognitive impairment, possibly by disrupting the endocannabinoid system in the hippocampus [97]. Clinical evidence indicates that long-term ethanol use can increase the levels of reactive oxygen species (ROS) and trigger a pro-inflammatory response, which may damage the BBB and contribute to neurodegeneration [98–101]. Even when alcohol consumption is stopped, the increased glutamatergic activity and oxidative stress also cause excitotoxicity and hyperactivity of glutamate receptors [102–104]. Notably, heavy alcohol consumption is associated with brain atrophy, neuronal loss and compromised white matter integrity [105]. Therefore, long-term excessive ethanol consumption may increase the risk of AD. Considering these factors, the disadvantages of ethanol therapy outweigh advantages and its potential therapeutic value still needs to be further evaluated.

#### Phenobarbital

Phenobarbital (C_12_H_12_N_2_O_3_) belongs to the barbiturate drug class and is a CNS depressant. It is a derivative of barbituric acid. It is a white and odorless powder, slightly soluble in water and alcohol. Phenobarbital does not exist in plants or animals and is always synthesized in the laboratory [106]. It is a positive allosteric modulator of GABA_A_R, thereby exerting sedative, anti-anxiety, hypnosis, anticonvulsant and muscle relaxation properties. Phenobarbital is mainly used for the treatment of epilepsy and insomnia [107]. Tau protein aggregation is a pathological hallmark of AD [4]. A previous study using a tau-biosensor-based cell high-throughput assay revealed that of seven anti-epileptic drugs, phenobarbital can significantly inhibit tau aggregation [108]. This provides a theoretical basis for anti-epileptic drug therapy in older adults with dementia. However, there is little literature on phenobarbital use for the treatment of AD, with most studies focusing on the treatment of AD complicated with epilepsy. Several clinical investigations have shown that people with AD are more likely to develop epilepsy, which may be related to neuronal hyperexcitability in the early stage of AD [109]. In patients with seizures and mild-to-moderate AD, phenobarbital worsens cognition and mood at 12 months of follow-up. However, the current evidence base is insufficient to reliably assess the efficacy and safety of phenobarbital in AD patients. Large randomized controlled trials with double-blind designs are needed to determine the efficacy and tolerability of phenobarbital in AD [110].

**Fig. 2:**
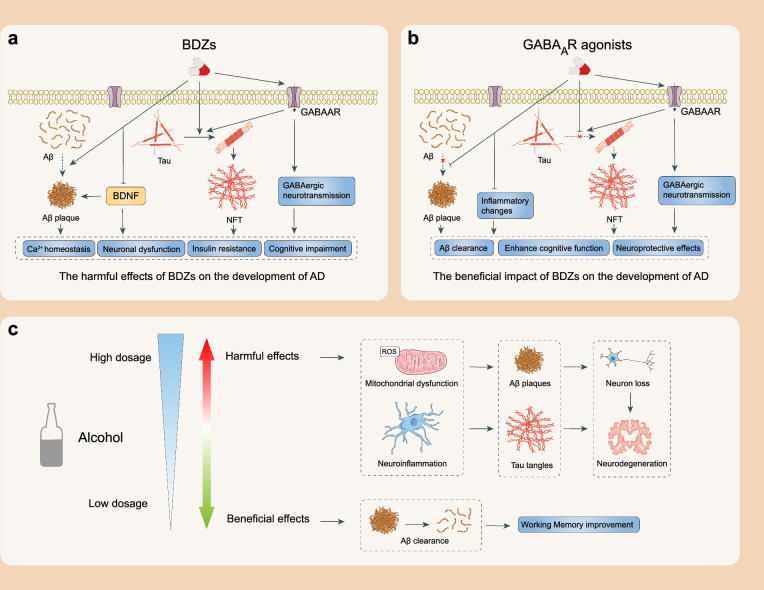
Schematic representation of the biphasic effects of CNS depressants. **a** Schematic showing mechanisms of action of BDZs in AD. BDZs function as positive allosteric modulators of GABA_A_R and enhance inhibitory GABAergic neurotransmission. BDZs cause calcium homeostasis imbalance, nerve damage, insulin resistance and cognitive impairment by enhancing Aβ and tau deposition, decreasing BDNF levels and over-activating GABAergic neurotransmission. **b** Schematic showing the mechanism and the superiority of GABA_A_R agonists for AD treatment. The GABAergic system is a potential target for blocking the pathophysiology associated with AD. GABA_A_R agonists play a neuroprotective role in AD by reducing Aβ toxicity, causing inflammatory changes and inhibiting tau protein phosphorylation. **c** Ethanol has bidirectional effects on AD. High doses of ethanol increase ROS and trigger pro-inflammatory responses, leading to neurodegenerative changes. Low doses of ethanol are beneficial for Aβ clearance and working memory improvement

The therapeutic potentials of different classes of CNS depressants for AD vary greatly. Specifically, BDZs show strong therapeutic effects against AD primarily by enhancing the function of the GABAergic system, which inhibits Aβ and tau protein aggregation. However, research on the use of ethanol and phenobarbital for AD treatment is very limited and their use often comes with certain health risks. Therefore, the value of ethanol and phenobarbital for treating AD is still highly debated and needs further investigation. Meanwhile, the adverse effects associated with long-term use of CNS depressants also need to be further studied to ensure their safety and efficacy (Fig. 2).

### Stimulants

Stimulants are drugs that increase CNS activity [111]. There are various types of stimulants, mainly caffeine, nicotine, and certain medications used to treat attention deficit hyperactivity disorder, such as methylphenidate (MPH) and amphetamines. Amino synthetic stimulants include cathinones, alkanes, phenethylamine, piperazine and tryptamine. Stimulants exert their effects mainly by increasing the levels of monoamine neurotransmitters serotonin (5-HT), dopamine and noradrenaline in the synaptic cleft [70, 112], either by stimulating the release of neurotransmitters from synaptic vesicles or by inhibiting the uptake of monoamine neurotransmitters [113, 114]. The increase of neurotransmitters in the synaptic cleft promotes activation of postsynaptic receptors, thereby improving the information processing capacity and the response speed of the brain. This is crucial for improving the cognitive function and daily activities of AD patients [115]. Such effect can not only enhance attention and memory, but also promote the release of neurotrophic factors, further supporting synaptic plasticity and neuroprotection. In the prefrontal cortex, the increase of neurotransmitters improves executive functions such as attention, decision-making and working memory [116]. In the hippocampus, the increase of neurotransmitters enhances the plasticity of synapses, thereby promoting the learning and memory processes. Furthermore, the increase of neurotransmitter concentrations in the synaptic cleft can trigger intracellular signaling pathways and provide neuroprotective effects [117].

#### Caffeine

Caffeine is a natural alkaloid and CNS stimulant found in coffee beans, tea leaves, cocoa beans and kola nuts. It blocks adenosine receptors and enhances alertness and cognitive function. High doses of caffeine can cause anxiety and insomnia [118, 119]. Some preclinical and epidemiological studies have suggested neuroprotective effects of caffeine on AD [120, 121]. Preclinical evidence has shown that caffeine intake mitigates Aβ-induced antioxidant activity and mitochondrial dysfunction [122–125], and inhibits enzymes for Aβ production [126–128]. A clinical study has reported that a lifetime coffee intake of ≥ 2 cups/day is significantly associated with lower Aβ positivity in the brain, further suggesting that caffeine has neuroprotective effects against AD [129]. However, a cross-sectional study indicates that caffeine consumption may be inversely associated with Aβ positivity in males but not females [130].

Adenosine receptor (AR) plays a critical role in the therapeutic efficacy of caffeine on AD. Adenosine is an endogenous neuroprotectant that is widely distributed in the CNS. Under conditions of brain injury, neuroinflammation and aging, adenosine concentration rises significantly [131]. Adenosine functions by interacting with G protein-coupled ARs, including inhibitory A1Rs and excitatory A2ARs [132]. Caffeine as a non-selective A2AR antagonist, has a similar purine base structure to adenosine. It competes with adenosine for A2AR binding and inhibits A2AR activation. This weakens the coupling of A2AR to G proteins and causes a cascade of downstream reactions such as adenylate cyclase activity and protein kinase A level decrease. These changes together regulate intracellular calcium overload, inhibit Aβ and tau protein aggregation and reduce oxidative stress and neuroinflammation, ultimately improving cognitive function [131, 133]. Acute caffeine administration prevents cognitive and memory dysfunctions in AD animal models [134]. Long-term caffeine administration also shows great therapeutic potential in alleviating AD pathologies such as Aβ aggregation and Aβ-induced neurotoxicity in mice [135–137]. It is important to note that the effect of caffeine on memory depends on its dose, timing and administration method. Other animal studies have shown that high doses of caffeine may have adverse effects on memory [138, 139].

#### Nicotine

Nicotine is an alkaloid found in solanaceae plants, mainly tobacco. As a CNS stimulant, it binds to nicotinic acetylcholine receptors (nAChRs) and enhances alertness and attention, but it may cause addiction [140]. Both preclinical and epidemiological studies have shown that nicotine has a positive effect on working memory-related cognitive function and is neuroprotective under certain conditions [141–144]. According to the cholinergic hypothesis, cognitive decline in AD is associated with deficits in cholinergic neurotransmission, primarily due to the loss of acetylcholine [145]. Animal studies indicate that nicotine activates α7 nAChRs [146, 147], thereby inducing the phosphorylation and activation of protein kinase B, a pivotal molecule in the PI3K/Akt signaling pathway [148, 149]. This pathway is involved in the regulation of neuronal function within the CNS, impacting processes related to neuronal survival, learning and memory [150–154]. Both acute and chronic nicotine administration alleviates cognitive impairment in AD patients. In AD patients treated with cholinesterase inhibitors, electroencephalogram readings show a shift toward normal levels following nicotine application [155–158]. Taken together, nicotine may alleviate mitochondrial dysfunction, ROS formation and Aβ- and tau-induced inflammation in AD patients through activating α7 nAChRs and the PI3K/Akt signaling pathway, ultimately improving learning and memory functions.

While nicotine replacement therapy has been confirmed as an efficient therapeutic intervention for AD due to its cholinergic effects, the long-term effects of nicotine use on AD remain uncertain [159]. Recent meta-analyses have demonstrated that nicotine smoking increases the risk of AD development [160, 161]. Overall, nicotine replacement therapy but not nicotine smoking may be a useful therapy for AD.

#### MPH

MPH is a CNS stimulant and synthetic drug. It does not exist naturally in plants or animals [162]. MPH exerts effects by binding to dopamine transporters (DATs) and noradrenaline transporters (NATs), non-competitively blocking the reuptake of dopamine and noradrenaline, thereby increasing the levels of these neurotransmitters in the synaptic cleft. Compared with amphetamine, MPH shows a stronger inhibitory effect on DAT and NAT and causes a more obvious enhancement of dopaminergic and noradrenaline transmission [163, 164]. Clinical studies have shown that MPH leads to a substantial increase of dopamine and noradrenaline levels in the striatum and prefrontal cortex. Furthermore, it protects the dopaminergic system from progressive "wearing off" by maintaining a large reservoir of neurotransmitters stored in presynaptic vesicles [165]. MPH has also been used to treat AD in recent years. Although some meta-analyses and systematic reviews suggest that MPH may be effective in treating apathy and certain aspects of AD [166], the conclusions remain inconsistent. A multicenter randomized placebo-controlled clinical trial has demonstrated safety and efficacy of MPH at a dose of 10 mg twice daily for the treatment of AD [167, 168]. However, another meta-analysis showed that MPH has no significant effects on the overall cognitive function at 6 months [169]. Therefore, more clinical and basic studies are needed to further evaluate the value of MPH in neurodegenerative disease treatment.

**Fig. 3:**
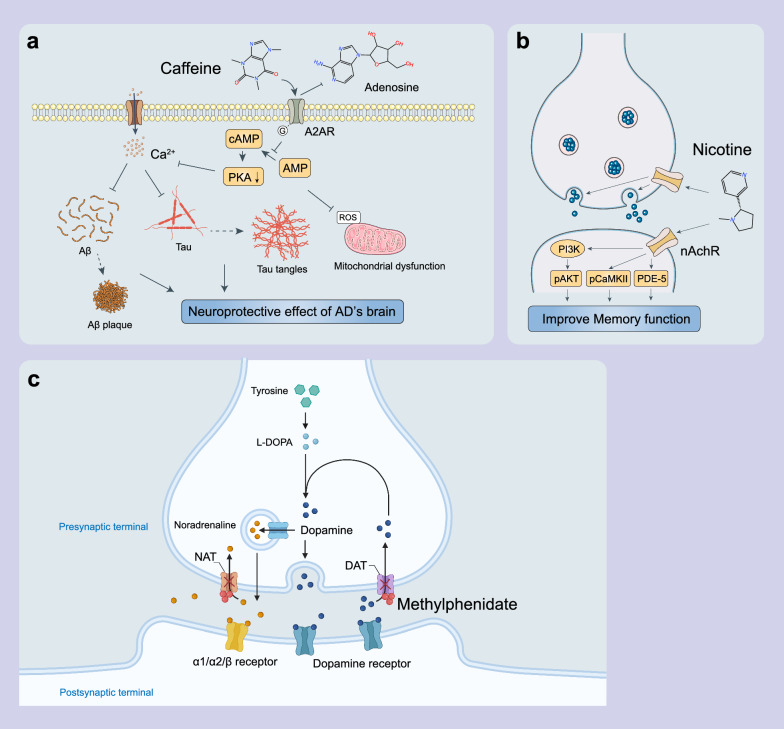
Schematic showing the mechanisms of CNS stimulants. **a** Caffeine effectively inhibits A2AR activation by competing with adenosine binding sites. This process decreases the conversion rate of AMP to cAMP and the level of PKA. **b** By activating nAChR and PI3K/Akt signaling pathways, nicotine may improve learning and memory and protect against neurological dysfunction and inflammatory processes caused by Aβ and tau deposition in AD. **c** Methylphenidate exerts its effects by non-competitively blocking the reuptake of dopamine and noradrenaline through interacting with DAT and NAT, and ultimately increases the levels of these neurotransmitters in the synaptic cleft. Figure 3c was created with BioRender.com/bt9p319

In conclusion, CNS stimulants present a complex and promising avenue for AD treatment through various mechanisms. The dose, timing and administration route should be delicately refined in clinical practice due to their influence on the efficacy of stimulants. Future research is needed to fully elucidate the underlying mechanisms to maximize the potential benefits while minimizing the risks. There is an urgent need to explore the long-term efficacy. These stimulants are potential anti-AD drugs that can improve the cognitive function and quality of life of AD patients (Fig. 3).

### Cannabinoids

Cannabinoids are widely used psychoactive substances. To date, more than 120 types of cannabinoids have been identified from cannabis plants, with tetrahydrocannabinol (THC) and cannabidiol (CBD) being the most common components. In addition, the marijuana leaf contains large amounts of cannabidiolic acid (CBDA) and tetrahydrocannabinolic acid (THCA), which are the acidic variants of CBD and THC, respectively. Anandamide (AEA) and 2-arachidonoylglycerol (2-AG) are critical endogenous agonists of CB_1/2_R, which have similar pharmacological effects to THC [170]. Synthetic cannabinoids are a chemically diverse group of cannabinoids that target the endocannabinoid system. Synthetic cannabinoids have greater potency and higher binding affinity to cannabinoid receptors compared to natural cannabinoids [171]. In recent years, cannabis and its compounds have been studied in the treatment of epilepsy, anxiety, AD and other neurological diseases [172, 173].

### *THC*

THC is the primary psychoactive compound in cannabis. It interacts with the G-protein-coupled CB_1/2_R in the endocannabinoid system, producing cognitive and euphoric effects. THC is naturally present in the cannabis plant and has potential therapeutic potency such as pain relief and appetite stimulation [174, 175]. CB_1_R is mainly localized at the axon shaft of neurons and regulates the release of presynaptic neurotransmitters, thereby participating in the regulation of various brain functions such as cognition, emotion, reward and memory [176]. CB_2_R is primarily distributed in glial cells and peripheral tissues [177]. THC has similar affinities for CB_1_R and CB_2_R, but most of the psychoactive effects of THC are attributed to CB_1_R activation [178].

Compared to currently approved drugs against AD, THC displays superiority in restraining Aβ aggregation [179, 180]. THC competitively inhibits acetylcholinesterase (AChE) and prevents the AChE-induced Aβ aggregation. Molecular modeling shows that THC binds to the peripheral anionic site of the AChE protein, a critical region contributing to Aβ formation. In a hydrogen peroxide-induced oxidative stress model, THC shows significant antioxidant activity and ameliorates AD-like pathological conditions, suggesting the potential of THC as an antioxidant [181].

While THC exhibits excellent therapeutic effects on AD, the routine use of high doses of THC may lead to serious psychiatric adverse effects [182, 183]. Currently, there is limited research on the efficient and safe dosages of THC for AD treatment. A study based on cell and transgenic mice suggests that low doses (0.02 and 0.2 mg/kg) of THC administered over three months significantly reduce the levels of Aβ oligomers, phosphorylated tau protein and total tau protein. It also increases the levels of Aβ monomers and phosphorylated GSK-3β, resulting in marked improvement in spatial learning in aged APP/PS1 mice in a dose-dependent manner [184]. Another study using 5 × FAD mice indicates that a single injection of ultra-low dose of THC ameliorates cognitive impairment in AD mice, by improving the balance between the truncated TrkB receptor subtype and the intact receptor [185]. These results suggest that low-dose THC may be a safe and effective therapeutic approach for AD.

Considering the multifactorial causes to neuronal degeneration in AD, development of new therapies needs to consider as many targets as possible. This is more effective than isolated use of a single cannabinoid compound. Studies have shown that combined use of THC and CBD decreases soluble Aβ42 levels, alleviating learning impairments and other AD-like phenotypes in APP/PS1 mice. This therapy also shows more pronounced anti-inflammatory properties compared to THC or CBD alone [186, 187]. Besides, a novel nanoscale formulation containing melatonin, insulin and THC has shown multi-targeted effects on Aβ formation, tau protein phosphorylation and mitochondrial dynamics, and improves spatial memory in aged APP/PS1 mice [188]. However, no synergistic effect is observed when caffeine and THC were administered together [189].

In addition to the natural cannabinoids, synthetic cannabinoid analogs, which are designed using molecular docking technology, also exhibit competitive inhibition of AChE and competitive antagonism of CB_1/2_R with reduced toxicity [190]. Therefore, phytocannabinoid-based analogs are a promising direction for developing novel therapeutic approaches against AD.

### *CBD*

Unlike THC, CBD is the dominant non-psychoactive component. It has a markedly reduced affinity for CB_1/2_R and usually acts as a negative allosteric modulator/inverse agonist of CB_1/2_R, thereby conferring a greater degree of safety for the treatment of AD [178, 186, 191–196]. CBD can spontaneously interact with tau monomers and inhibit the formation of tau tangles [197]. CBD can also inhibit Aβ aggregation and preserve synaptic plasticity in AD [198, 199]. Behavioral studies based on APP × PS1 mice suggest that CBD reverses Aβ-induced spatial memory deficits and social recognition impairments without affecting anxiety-related behaviors [194, 200, 201]. Magnetic resonance imaging of human samples shows that CBD increases cerebral blood flow in key regions associated with memory processing, particularly in the hippocampus [202].

Recent studies have highlighted the potential of CBD in the treatment of AD. CBD ameliorates cognitive decline by potentiating the function of extra-synaptic glycine receptor (GlyR) in the 5 × FAD mouse model of AD. This finding underscores the significant role of CBD in reducing neuronal hyperexcitability through modulating GlyR function [203]. GlyR, a pentameric ligand-gated chloride channel responsible for maintaining the excitatory/inhibitory balance in the CNS, is particularly prevalent in the hippocampus and cortex [204–208]. By enhancing GlyR function, CBD has therapeutic potential for neurological disorders characterized by hyperexcitability, such as epilepsy and the rare startle syndrome [209–214]. Therefore, CBD may alleviate abnormal neuronal excitability and reduce Aβ deposition in the hippocampus of AD patients by potentiating the function of GlyR. Drug-target affinity prediction models have identified six potential CBD targets associated with AD, including endothelial nitric oxide synthase, disintegrin and metalloproteinase 10 myeloperoxidase, APOE, APP and PSEN1. CBD also acts as a reverse agonist at GPR3 and inhibits the recruitment of β-arrestin2 to GPR3 [215, 216].

CBD can also exert neuroprotective and anti-inflammatory effects by inhibiting Aβ-induced microglial activation [217]. By activating transient receptor potential vanilloid 2 (TRPV2), CBD enhances microglial phagocytosis of Aβ and mRNA expression of phagocytic receptors, thus reducing neuroinflammation and improving mitochondrial function [195, 218]. Furthermore, CBD scavenges intracellular ROS and inhibits lipid peroxidation without inducing overexpression of antioxidant genes. In addition, CBD increases the organism's resistance to oxidative stress, which is independent of the classical transcription factors DAF-16 and SKN-1. This suggests that the in vivo antioxidant activity of CBD is probably due to its intrinsic antioxidant properties [180, 198]. CBD treatment significantly increases the expression of interleukin (IL)−33 and triggers expression of its receptor on myeloid cells, improving cognitive function in 5 × FAD mice [219].

**Fig. 4:**
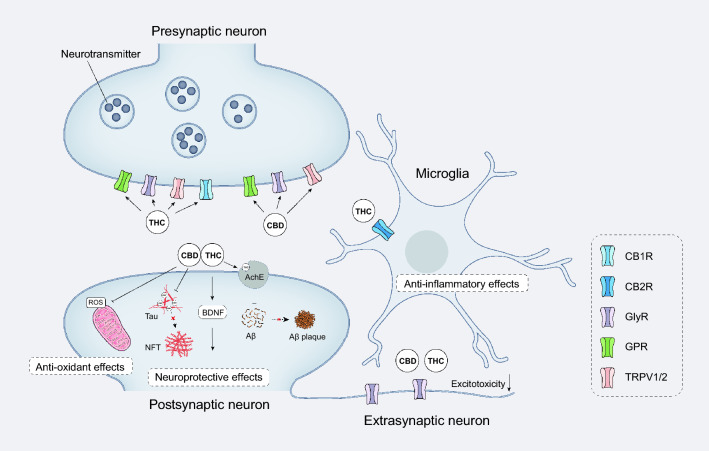
Schematic of the neuroprotective effects of THC and CBD. While THC has a similar affinity for CB_1/2_R and its psychoactive effects are primarily associated with CB_1_R activation, CBD has a very low affinity for CB_1/2_R and tends to act on GlyR and TRPV. THC and CBD inhibit Aβ aggregation, tau protein phosphorylation and neuronal hyperexcitation, showing excellent therapeutic effects on AD

In summary, CBD can ameliorate pathological processes and reverse cognitive deficits in AD through interaction with various AD targets such as microglia, antioxidant and anti-inflammatory properties, mitochondrial dysfunction, tau protein aggregation and neuron hyperexcitability. THC exerts anti-AD activity mainly by targeting CB_1/2_R, which may cause serious psychiatric adverse reactions. CBD exerts its effects by interacting with other membrane proteins especially GlyR. Therefore, CBD is safer and shows great value for AD treatment (Fig. 4).

#### Phytocannabinoids

Some other phytocannabinoids such as cannabichromene (CBC), cannabigerol (CBG), cannabinol (CBN) and cannabidivarin (CBDV) also show great protective properties against neurodegenerative disease. Studies at the cellular level have shown that CBC, CBG, CBN and CBDV can inhibit Aβ42-induced neurotoxicity and block Aβ aggregation [180]. Cannflavin A reduces Aβ-mediated neurotoxicity and inhibits Aβ fibrillisation [220]. Both CBDA and THCA reduce the levels of Aβ and phosphorylated tau, alleviate calcium dysregulation and finally reverse memory loss in AD [221]. However, the detailed molecular mechanisms remain to be fully understood. Systematic studies regarding the interacting mechanism of these phytocannabinoids with various neurotransmitter receptors and how they specifically regulate neuroprotective signaling pathways are still lacking. Therefore, future studies need to focus on elucidating the neuronal mechanism and targets of these phytocannabinoids, in order to develop more avenues for the treatment of AD.

#### AEA

AEA is a fatty acid neurotransmitter and an endogenous cannabinoid present in the human body and in foods such as chocolate and truffles. It binds to CB_1/2_R and affects mood, appetite and memory. AEA is known as the molecule of Bliss, which increases during exercise and enhances feeling of happiness [222]. Clinical evidence shows that AEA and its precursor N-arachidonoyl phosphatidylethanolamine are significantly reduced in the frontal and temporal cortex of AD patients. Such reduction is correlated with increased levels of neurotoxic Aβ42 protein [223]. AEA acts as an endocannabinoid and is degraded by the enzyme fatty acid amide hydrolase (FAAH). In the frontal cortex of AD patients and animal models, FAAH activity has been shown to be reduced. Consistently, FAAH genetic inactivation improves the memory deficits in 5 × FAD transgenic mice [224, 225]. Structural analogs of AEA also exhibit neuroprotective effects by intervening in multiple pathological processes to slow down the deteriorating process of AD. For instance, the AEA analog N-stearoylethanolamine improves spatial memory in AD model mice via reducing Aβ and tau aggregation [226].

#### 2-AG

2-AG is a key endocannabinoid, a glycerol lipid molecule acting as a signaling compound in the endocannabinoid system. As an endogenous agonist of CB_1/2_R, it regulates various physiological processes. It is synthesized from precursor lipids in cell membranes and is widely distributed in the brain and other tissues. 2-AG synthesis is linked to demand and it is deactivated by the enzyme monoacylglycerol lipase (MAGL) [222, 227]. 2-AG acts as an endogenous anti-inflammatory agent through a CB_1_R-mediated mechanism, which contributes to the maintenance of brain homeostasis [228, 229]. Numerous studies have shown that 2-AG protects against Aβ-induced neurodegeneration and apoptosis of primary hippocampal neurons and these effects can be inhibited by CB_1_R antagonists [229, 230]. MAGL is the principal enzyme that degrades 2-AG in the brain. MAGL-mediated hydrolysis of 2-AG terminates the activation of CB_1/2_R, releasing arachidonic acid and glycerol in the brain. Pharmacological or genetic inhibition of MAGL significantly increases 2-AG concentrations and alleviates Aβ-induced neurodegeneration and apoptosis, improving synaptic function and cognitive function in AD [230–234]. These findings support that MAGL is a promising therapeutic target for developing safe and effective anti-AD agents.

#### Potential values and challenges of cannabinoids for AD treatment

**Fig. 5:**
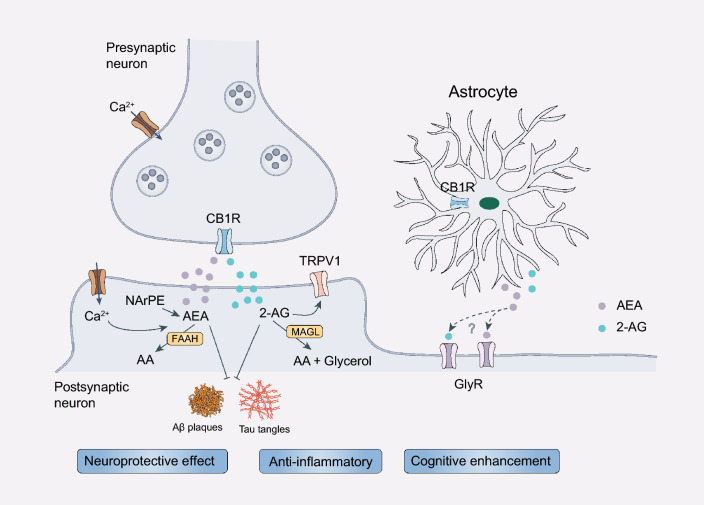
Schematic of the neuroprotective effects of AEA and 2-AG. Endocannabinoids are synthesized based on the demand of the body. Their concentrations are regulated by decomposition. Endocannabinoids have modulatory effects on CB_1_Rs and TRPV1, which exert anti-inflammatory and neuroprotective effects

Currently, most of the evidence for the protective effects of cannabinoids against AD was derived from in vitro and in vivo animal models. Furthermore, considering the heterogeneity of cannabis components and various adverse effects on mental health, future research should focus on the development of multi-target strategies and novel cannabinoid analogues to improve the therapeutic efficacy and reduce side effects (Fig. 5).

However, the clinical use of cannabis and its derivatives faces significant legal and regulatory challenges. The legal status of cannabis varies widely across jurisdictions, affecting its availability for research and treatment [235]. Different cultivation and production standards can lead to variability in cannabinoid concentrations and purity, complicating quality control efforts [236]. In addition, patients may encounter obstacles in accessing cannabis-based treatment due to restrictive regulations or lack of insurance coverage. Education should be provided to health professionals regarding the therapeutic potential and safe use of cannabis. In addition, future studies are needed to develop multi-target strategies and novel cannabinoid analogues with improved therapeutic efficacy and reduced side effects while addressing these regulatory challenges.

### Psychedelics

The term “psychedelic” can be traced back to the latter half of the twentieth century. Psychedelics are a group of substances that can change or enhance sensory perceptions, thought processes and energy levels. Due to their potential capacity for inducing imaginary perceptions, they are always referred to as “hallucinogens” [237]. Although some psychedelics are extracted directly from natural plants such as psilocybin and mescaline, most of them are chemically synthesized. Based on their chemical structure (including an aromatic group, an alkaline amine and a dicarbon linker), psychedelics can be classified into two classes: (1) tryptamines characterized by C3-substituted indole rings such as bufotenine, psilocin and D-lysergic acid diethylamide (LSD); and (2) phenethylamines that possess a phenyl group such as mescaline [237]. This structural configuration is critical for the binding of psychedelics to receptors [238]. Serotonin 2 A receptor (5-HT_2A_R) is the representative psychedelics receptor [239] and can be activated by most psychedelics, leading to increased neuronal activity and enduring neural structural alterations [240]. However, the downstream effects may be further modulated by various additional factors such as the relative density and subcellular distribution of subtypes of receptors, as well as the psychedelic dosage [241].

Psychedelic drugs offer potent tools for exploring the human mind and have shown significant benefits for individuals with neuropsychiatric disorders like depression, anxiety, and substance-use disorders [242–246]. Despite a decade-long research hiatus due to controlled substance laws, recent years there has been a resurgence of scientific interest in psychedelics, especially their potential for treating AD [247, 248].

#### *N*,* N*-dimethyltryptamine (DMT)

DMT is a natural indole alkaloid extracted from numerous plant species and botanical preparations. DMT is a hallucinogenic compound that mediates cognition and perception in humans by targeting the 5-HT_2A_R [249, 250]. The sigma-1 receptor is another important downstream element of DMT in the CNS [251, 252]. DMT significantly reduces microglial density in the AD model established by intracerebroventricular injection of Aβ42 oligomers, but fails to restore the activated state of microglia. Meanwhile, DMT inhibits the reactivation of astrocytes induced by Aβ_42_ and leads to decreased expression of tumor necrosis factor-α and increased expression of sigma-1 receptors. Sigma-1 receptors are ligand-regulated molecular chaperones that function to inhibit various voltage-sensitive ion channels. Thus, the psychedelic effects of DMT may be mediated in part through sigma-1 receptors [253]. Higher concentrations of DMT lead to inhibition of voltage-sensitive ion channels by triggering the transfer of sigma-1 receptors from the mitochondria-associated endoplasmic reticulum to the plasma membrane. More importantly, activation of sigma-1 receptors by agonist 2-(4-morpholinethyl)−1-phenylcyclohexanecarboxylate leads to significant reduction of Aβ-triggered neuronal death [254]. These findings not only demonstrate the efficacy of DMT in ameliorating Aβ-induced neuroinflammation, but also highlight sigma-1 receptors as a promising target for developing novel anti-neuroinflammatory drugs against AD. Nevertheless, the therapeutic potential of DMT in AD remains to be confirmed in clinical studies and the mechanisms underlying the effects on cognitive function remain to be explored in future studies.

#### LSD

LSD is a potent synthetic hallucinogenic drug derived from ergot fungus. It mainly interacts with the 5-HT_2A_R and mediates perception and cognition. LSD is mainly chemically synthesized. LSD was very popular in psychotherapy in the 1960 s and is currently used in research of depression and anxiety disorders [255, 256]. Although LSD is proven to be a powerful and effective anxiolytic and antidepressant drug, it shows no cognitive benefits in AD animals and patients [257, 258]. Both Aβ and neuroinflammation in AD can be modulated by psychedelics through the 5-HTergic system. On the one hand, inhibiting 5-HT reuptake decreases Aβ production and total Aβ amounts in brains of transgenic AD model mice [259]. On the other hand, the activity of the 5-HTergic system is reduced [260], and knocking out 5-HT_2B_R causes significant morphological changes in microglia within AD animal model [248, 261, 262], suggesting that psychedelics may improve AD symptoms through the 5-HTergic system and more evidence is needed.

**Fig. 6:**
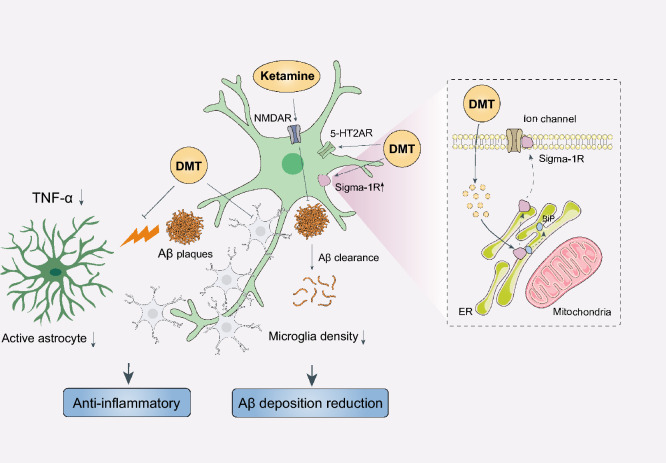
Schematic of the beneficial effects of psychedelics and ketamine. Most psychedelics such as DMT have a high affinity to 5-HT_1/2_R, especially the 5-HT_2A_R. In addition, DMT can reduce Aβ-induced neuroinflammation through sigma-1 receptors. Higher concentrations of DMT inhibit voltage-sensitive ion channels by triggering the transfer of sigma-1 receptors from the mitochondria-associated endoplasmic reticulum to the plasma membrane. Ketamine exerts anti-inflammatory and neuroprotective effects by acting on NMDAR

The therapeutic mechanisms of psychedelics on AD are more intricate than previously assumed and involve more than the 5-HT pathway. The sigma-1 receptors are also a critical point since their agonists significantly ameliorate cognitive decline in AD [263], accompanied by reduction of Aβ42 level, attenuation of microglial activation and suppression of inflammatory factors. Psychedelic substances, including LSD, ketamine and DMT, can modulate neuronal plasticity through augmentation of long-term potentiation and exhibit anti-inflammatory and neuroprotective properties by promoting the activation of the mTOR/AKT signaling cascade [240]. The common mechanisms underlying psychedelics should be investigated in future, to accelerate the development of safe and effective anti-AD drugs (Fig. 6).

### Opioids

Opioids are derived from opium poppy plants and cover more than 20 subtypes. Of these subtypes, morphine and codeine are the most widely used analgesics [70]. Opioids can be divided into several classes based on their chemical structure: chemicals of the 4,5-epoxide morphine ring (e.g., morphine), the phenylpiperidines (e.g., fentanyl) and the diphenylheptamines class (e.g., methadone). Synthetic opioids are always chemically modified from these base compounds [264]. Opioids and synthetic opioids act on the same types of receptors in the brain and elicit similar physiological responses, including pleasure, anxiety reduction, relaxation and sleepiness. Opioids, as partial or total agonists of G protein-coupled opioid receptors in the CNS, can activate δ, κ and μ receptor subtypes. The μ-opioid receptors are the primary site of action of opioids to produce core pharmacological effects including euphoria, analgesia, respiratory depression and drug dependence [265].

Opioids are widely used in clinical practice as potent analgesics, but their abuse has deleterious effects on cognitive function through accelerating tau protein hyperphosphorylation and disrupting neural stem cell homeostasis [266–268]. Morphine, the most frequently used opioid in the clinic, enhances the activity of P450 cytochrome aromatase and facilitates the release of estradiol from hippocampal neurons. This then induces up-regulation of heat shock protein 70, which rescues the intracellular proteasome activity from Aβ toxicity [269]. Morphine also exerts protective effects by regulating Aβ metabolism through the nitric oxide pathway [270]. While opioids have the potential to regulate certain pathological processes, these effects fail to translate to therapeutic benefits in AD due to their strong association with cognitive impairment, hyperphosphorylated tau protein and neuroinflammation [271, 272].

Considering the high risk of addiction and lethality of overdose opioids, the use of opioids in the treatment of chronic neurodegenerative diseases such as AD must be made with great caution, with rigorous evaluation of safety in patients. There is currently very little evidence on the clinical efficacy and safety of opioids for the treatment of AD [273, 274]. Therefore, more preclinical studies and large-scale trials are needed to assess the possibilities and safety of opioids in treating AD.

### Ketamine

Ketamine is a fast-acting general anesthetic that works by blocking NMDA receptors. Although developed for anesthetic purposes initially, it was later found with potential for therapeutic and recreational uses. Ketamine is particularly effective for rapid induction of anesthesia, which explains its common use in emergency medicine and pediatric settings. When taken at low doses, ketamine causes a feeling of being dissociative from the body and environment, always accompanied by visual and auditory hallucinations and the sensations of being outside the body [275].

Ketamine has emerged as a treatment option for depression, especially in cases where other treatments have failed, owing to its rapid antidepressant properties [276–280]. Notably, it has anti-inflammatory and neuroprotective effects by antagonizing NMDA receptors in the CNS, suggesting its potential use in AD patients [281]. For instance, ketamine significantly reduced the number of Aβ_42_-positive neurons in aged rats and improved their cognitive performance in novel object recognition and Morris water maze tests [282]. However, some studies showed that ketamine impairs cognition. In a recent study, chronic ketamine exposure leads to cognitive impairments by reducing the expression of glutamate receptors, the dendritic spine density and synaptic transmission in mice, which is mediated through inhibition of expression and phosphorylation of CaMKIIβ, ERK1/2, CREB and NF-κB [283]. Another study in 24 healthy young adults reported that *S*(+)- and *R*(−)-ketamine induce less cognitive impairment than racemic ketamine, and that *S*(+)-ketamine causes less decline of memory compared with *R*(−)-ketamine [284]. Furthermore, fMRI-based evidence involving 11 frequent ketamine users and 15 poly-drug controls indicates that long-term ketamine use is also associated with spatial memory impairments and medial temporal lobe dysfunction [285]. Therefore, the therapeutic potential of ketamine in AD remains controversial and requires further research.

Recently, research focus has shifted towards the ketamine metabolite (2R,6R)-hydroxynorketamine (HNK), which has demonstrated beneficial effects in AD models [286]. In Aβ oligomer-infused mice, HNK activates ERK1/2 (extracellular signal-regulated kinase 1/2), mTOR (mechanistic targets of rapamycin) and p70S6 kinase 1/ribosomal protein S6 signaling pathways, which are crucial for protein synthesis and memory consolidation. HNK also rescued synaptic plasticity and memory, mainly through ERK signaling. HNK also corrected aberrant transcriptional signatures in aged APP/PS1 mice, suggesting a potential therapeutic approach in AD [286].

## Conclusion, challenges and future directions

The exploration of psychoactive substances in AD treatment is a complex landscape, with each class of compounds exhibiting distinct mechanisms of action and therapeutic potentials. While some psychoactive substances show promise in modulating neurophysiological functions and enhancing cognitive performance, it is necessary to recognize the heterogeneity within this population and the varying degrees of benefits and risks they present.

CNS depressants are mainly represented by BDZs in AD treatment. BDZs improve cognitive function by activating GABAergic neurons and inhibiting Aβ accumulation, but long-term use can cause memory problems and cognitive impairment. Although the relationship between BDZs and AD is controversial, research supports the GABAergic system as a potential target for the treatment of AD. Future studies should further explore the role of GABAergic systems in AD and develop new therapies.

Similarly, stimulants like caffeine and nicotine have shown potential to alleviate AD symptoms, but their long-term effects and optimal therapeutic windows need to be carefully defined. Cannabis-derived compounds, including THC and CBD, have shown potential to regulate Aβ aggregation and tau protein phosphorylation. However, the clinical use of cannabis and its derivatives faces legal and regulatory challenges. In addition, developing standardized protocols for the use of cannabinoids in clinical settings is critical for patient safety and accurate evaluation of its efficacy in AD. Psychedelics such as LSD and DMT, have shown intriguing results in regulating neuroplasticity and inflammation, but their use in AD treatment is still largely experimental and requires rigorous clinical validation.

Psychoactive substances such as ethanol and opioids show complex effects in AD. While moderate ethanol consumption may have some protective effects against Aβ-induced synaptic failure, there is a lack of established safe consumption levels. The risks associated with long-term use, including neuroinflammation and an increased risk of AD, must be carefully considered. Additionally, the combination of ethanol and opioids causes serious health risks such as respiratory depression and potential overdose. Opioids are effective analgesics but can also cause cognitive decline and hyperphosphorylation of tau protein, which has raised concerns about their use in AD patients. Although they may provide some neuroprotective effects, these benefits are always outweighed by the potential addiction and adverse cognitive outcomes. In conclusion, both ethanol and opioids have potential benefits but also significant risks and limitations in AD treatment. A careful evaluation of their pharmacological effects is essential for developing safe and effective therapeutic strategies for AD.

In view of the diverse effects of psychoactive substances, an advanced understanding of their molecular mechanisms and clinical significance is critical for optimizing therapeutic potential while minimizing adverse effects.

## Data Availability

Not applicable.

## Abbreviations

AD: Alzheimer's disease
CNS: Central nervous system
Aβ: Amyloid-beta
NFT: Neurofibrillary tangles
APOE: Apolipoprotein E
APP: Amyloid precursor protein
PSEN: Presenilin
GABA_A_R: Gamma-aminobutyric acid type A receptor
BDZs: Benzodiazepines
THC: Δ9-Tetrahydrocannabinol
CBD: Cannabidiol
THCA: Tetrahydrocannabinolic acid
CBDA: Cannabidiolic acid
AEA: Anandamide
2-AG: 2-Arachidonoylglycerol
CBC: Cannabichromene
CBG: Cannabigerol
CBN: Cannabinol
CBDV: Cannabidivarin
DMT: *N*, *N*-dimethyltryptamine
LSD: Lysergic acid diethylamide
NMDA: *N*-methyl-d-aspartate
GABA: Gamma-aminobutyric acid
ROS: Reactive oxygen species
BBB: Blood–brain barrier
GlyR: Glycine receptor
TRPV2: Transient receptor potential vanilloid 2
IL: Interleukin
FAAH: Fatty acid amide hydrolase
MAGL: Monoacylglycerol lipase
mTOR: Mechanistic target of rapamycin
nAChRs: Nicotinic acetylcholine receptors
AChE: Acetylcholinesterase
5-HT: 5-Hydroxytryptamine (serotonin)
5-HT_2A_R: Serotonin 2A receptor
5-HT_2B_R: Serotonin 2B receptor
HNK: Hydroxynorketamine
DAT: Dopamine transporter
NAT: Noradrenaline transporter
MPH: Methylphenidate
MST1: Macrophage stimulating 1
